# Placental CRH as a Signal of Pregnancy Adversity and Impact on Fetal Neurodevelopment

**DOI:** 10.3389/fendo.2021.714214

**Published:** 2021-08-02

**Authors:** Ifigeneia Kassotaki, Georgios Valsamakis, George Mastorakos, Dimitris K. Grammatopoulos

**Affiliations:** ^1^Department of Internal Medicine, 2nd Internal Medicine Clinic, Venizeleio Pananeio General Hospital, Heraklion, Greece; ^2^Second University Department of Obs and Gynae, Aretaieion Hospital, Medical School, National and Kapodistrian University of Athens, Athens, Greece; ^3^Translational Medicine, Warwick Medical School, Coventry, United Kingdom; ^4^Unit of Endocrinology, Diabetes Mellitus and Metabolism, Aretaieion Hospital, Medical School, National and Kapodistrian University of Athens, Athens, Greece; ^5^Institute of Precision Diagnostics and Translational Medicine, Pathology, University Hospitals Coventry and Warwickshire (UHCW) NHS Trust, Coventry, United Kingdom

**Keywords:** CRH, fetal, neurodevelopment, maternal stress during pregnancy, CRH receptor-1, inflammation

## Abstract

Early life is a period of considerable plasticity and vulnerability and insults during that period can disrupt the homeostatic equilibrium of the developing organism, resulting in adverse developmental programming and enhanced susceptibility to disease. Fetal exposure to prenatal stress can impede optimum brain development and deranged mother’s hypothalamic–pituitary–adrenal axis (HPA axis) stress responses can alter the neurodevelopmental trajectories of the offspring. Corticotropin-releasing hormone (CRH) and glucocorticoids, regulate fetal neurogenesis and while CRH exerts neuroprotective actions, increased levels of stress hormones have been associated with fetal brain structural alterations such as reduced cortical volume, impoverishment of neuronal density in the limbic brain areas and alterations in neuronal circuitry, synaptic plasticity, neurotransmission and G-protein coupled receptor (GPCR) signalling. Emerging evidence highlight the role of epigenetic changes in fetal brain programming, as stress-induced methylation of genes encoding molecules that are implicated in HPA axis and major neurodevelopmental processes. These serve as molecular memories and have been associated with long term modifications of the offspring’s stress regulatory system and increased susceptibility to psychosomatic disorders later in life. This review summarises our current understanding on the roles of CRH and other mediators of stress responses on fetal neurodevelopment.

## Introduction

Early life, especially the first 1000 days from conception to age 2, is considered as one of the most critical periods of development (1), where the foundations of optimum health, growth, and neurodevelopment across the lifespan are established. During pregnancy, the mother must activate and coordinate multiple and diverse homeostatic mechanisms to support the growing fetus, especially neurodevelopment and achieve a favourable outcome. Optimal function of this adaptation process (2), involving mediators of the neuroendocrine stress response, promotes brain plasticity and resilience. In cases of excessive prenatal adversity, optimal homeostatic equilibrium might be severely impaired (3) and (mal)adaptive responses might contribute to development of prolonged pathogenic mechanisms. Pathological signals arising from the mother can be transmitted to the developing fetus leading to adverse programming of the offspring (2, 4).

Altered developmental trajectories of the fetus, can lead to acute consequences or long-term outcomes such as enhanced susceptibility to adult disease (the fetal origin of adult disease) (5). A plethora of studies identified correlations between disrupted fetomaternal symbiosis and fetal programming sometimes leading to pathological birth phenotypes including abnormal immune function (6, 7) and increased metabolic risk of the offspring (8–10). Prenatal stress has also been associated with disrupted brain programming and function, since the perinatal period is a critical period of neurogenesis where the fetal brain can be remodelled or re-programmed (11). Such exposures to adverse early life experiences that disrupt fetal neurodevelopment are associated with offspring’s increased risk for various psychopathologies (12–15).

In this review we describe current knowledge and emerging evidence about the key players involved in maternal stress responses on fetal neurodevelopment, focusing on the two distinct but interacting mediators of hypothalamic–pituitary–adrenal axis (HPA axis) responses: corticotropin-releasing hormone (CRH) and its G-protein coupled receptors (GPCRs), and adrenal glucocorticoids (GCs).

## Adaptation to Maternal Stress, Resilience, and Neurodevelopment

In human, the period between 20 and 32 weeks after conception is characterized by rapid brain development in particular neural migration and synaptogenesis and a high rate of fetal neuronal proliferation (16); this is associated with development of the fundamental anatomical structures for the initial functioning of early neural circuits *in utero* (17). Brain neurogenesis is remarkably complex and the fetal brain tissue can be particularly plastic and vulnerable to a hostile intrauterine environment. During pregnancy, coordinated actions of hormones produced by the mother, placenta and fetus, regulate fetal neurodevelopmental processes and fine-tune brain formation (18). Members from at least three families of GPCRs, (rhodopsin, secretin and adhesion) have been identified as crucial for mediating these actions (18–20) (**Table 1**).

**Table 1:** GPCRs involved in CNS development.

Receptor family	Ligand example	Type of receptor	Actions on neuronal tissue
**Secretin GPCRs**	CRH	CRH-R1	Neurogenesis, differentiation of neuronal cells, development of neuronal circuits (21, 22)
**Rhodopsin GPCRs**	Serotonin	5-HT_1A_, 5-HT_2A_, 5-HT_2C_	Development of neuronal circuits Modulation of memory, emotions and cognition (20)
	Oxytocin	OT-R	Myelination, anti-inflammatory actions, neuroprotection (18)
**Adhesion GPCRs**			Architecture and wiring of cortical and subcortical brain areas (19)

Fetal exposure to maternal stressors and enhanced allostatic load, can disrupt optimum brain development. Mapping pathologic pathways implicated as central mediators of the effects of early life stress in brain function, identified altered HPA axis responses and perturbed glucocorticoid signalling as well as various modifications in the fetal brain function, including impaired GPCR signalling, alterations in neurotransmission and disturbed functionality of neuronal circuits (21–26). Thus, as the phenotype responds to the intrauterine environment, these adaptations can sometimes result in long term consequences and increased risk for disease in later life (27). See Howland et al. (28) for a recent review.

### HPA Axis Activation and Pathological Offspring Phenotypes

The HPA responses during pregnancy are characterized by another major source of neuropeptide secretion, the placenta, which synthesises placental CRH (pCRH) as early as 7 weeks of gestation. Placental CRH exhibits distinct responses to glucocorticoids and during gestation there is a bi-directional release of pCRH into the maternal and fetal compartments (29, 30). Acting *via* type 1 (CRH-R1) and type 2 (CRH-R2) receptors, CRH coordinating homeostatic challenges (31) that might be crucial in fetal maturation and providing nutritional signals that ultimately control pace of fetal development. It is thus reasonable to assume, that hormonal imbalances associated with severe or prolonged stress response, could potentially affect optimal outcomes (18). For example, it has been suggested that prenatal maternal stress signals associated with elevated levels of CRH, influence fetal growth in directions that determine gestation outcomes and alter birth phenotypes (32). Results from the ELGAN (Extremely Low Gestational Age Newborns) study suggest that extremes of pCRH expression identify risk for adverse fetal developmental outcomes: low CRH mRNA concentrations are associated with placenta inflammation and predict ventriculomegaly whereas high CRH mRNA concentrations predict motor dysfunctions (33). In addition, pCRH through cortisol stimulation might induce a state of insulin resistance and increased glucose in maternal circulation available to the fetus.

### CRH as a Neuroprotective Signal and Molecular Mechanisms

A potential neuroprotector role has been suggested for CRH by promoting neurogenesis, differentiation and survival of neuronal cells (34, 35), however, abnormal intrauterine exposure to excess CRH, can affect fetal neurodevelopment and result in brain alterations resulting in cognitive and emotional deficits that persist in later life (16, 36–39). Studies on early human embryos suggest that CRH can promote survival of the neural progenitor cells (NPCs) and serve as an endogenous neuroprotector. These actions involve CRH-R1 and downstream activation of multiple kinases including PKA and CREB activation (40) as well as MAPK and PI3K signalling pathways. The latter control apoptosis of NPCs, through inactivation of proapoptotic signals such as Glycogen Synthase Kinase (GSK-3β) that prevents degradation of β-catenin, augmenting neurogenesis (41). CRH direct neuroprotective actions can oppose the neurotoxic effects of excess glucocorticoids on neuronal progenitors (34). Additionally, CRH serves as a key modulator in adult neurogenesis and genetic disruption of CRH/CRH-R impairs hippocampal neurogenesis. Exposure of hippocampal neural stem cells (NSCs) to CRH increases proliferation, survival, and differentiation *via* transcriptional processes involving upregulation of Notch3, a crucial regulator of adult tissue NSC quiescence and maintenance (35).

CRH control of neurogenesis involves regulators of neuronal connectivity and synaptic plasticity such as brain derived neurotrophic factor (BDNF). Hypothalamic CRH is positively regulated by BDNF *via* a mechanism that involves the cAMP response-element binding protein (CREB) coactivator CRTC2. This transcriptional regulator serves as a bidirectional switch for BDNF and glucocorticoids to control expression of CRH (42). Studies in CRH-overexpressing mice also identified a positive feedback loop between CRH and BDNF that enhances BDNF release. This leads to improved neuroprotective outcomes under acute excitotoxic stress, with reduced neurodegeneration and neuroinflammation of the hippocampus (43). Stress-induced epigenetic modifications, resulting in BDNF methylation and decreased expression in prefrontal cortex, amygdala and hippocampus of prenatally stressed rats (44, 45), disrupt the optimum neurodevelopmental processes, with similar effects also reported in humans (46).

### Fetal Neurodevelopment in States of Maternal Stress and Excess CRH

Placental CRH can cross the immature fetal blood-brain barrier where it may alter the rate of maturation of developing neuronal structures. Differentiated cortical neurons in the fetal brain express CRH-R as early as 13 weeks’ gestation. Studies in humans and animals linking pCRH with early life behavioural outcomes, show a positive correlation between maternal stress and aberrant neurodevelopmental function likely related to stunting of normal neuronal growth. See Lautarescu et al. (47) for a recent review. Reduced cortical volume in the frontal and temporal lobe, in the face of elevated levels of pCRH during gestation, have been associated with both cognitive and emotional deficits in pre-adolescent children (36). Moreover, in rodents models of disease, stress-induced dendritic remodelling has been linked with increased anxiety and depressive like behaviours and with memory impairment (48). In human studies, fetal exposure to accelerated pCRH trajectories during mid-gestation was associated with child internalizing symptoms at 5 years of age (16) and prenatal exposure to high pCRH can affect infant temperament. Full term infants of mothers with lower CRH levels at 25weeks of gestation showed lower levels of distress in infancy compared with infants exposed to elevated levels of the stress hormone (49).

Placental CRH levels have been positively correlated with thinning of selective brain regions during gestation. The impact of such developmental effects on maturation of neurons and brain circuits is long-lasting as it is evident into early life: children exposed to elevated levels of pCRH prenatally exhibit significant thinning in the whole cortical mantle at age 7. The timing of the exposure to altered pCRH levels also determines region specific changes; prefrontal thinning is associated with elevated pCRH levels at early gestation whereas temporal thinning is associated with pCRH levels later in gestation (36). Experimental animal models demonstrated CRH-induced alterations in dendritic brunching, specifically, decreased branching of cortical neurons (37); altered synaptic plasticity, impaired myelin formation and decreased dendritic spine density in the hippocampal region of the offspring (50, 51). Some evidence link maternal anxiety to reduced fetal amygdala volume during the late second and third trimester of pregnancy and alterations in fetal cortical gyrification of the frontal and temporal lobes in brains of human fetuses of stressed mothers (52).

The hippocampus and the HPA axis are functionally interconnected, therefore stress alterations to the HPA axis could mediate changes in the developing hippocampus (4). Recent studies demonstrated that maternal adversity involves brain CRH-R1 activation and regulation of neuronal connectivity and developmental trajectories of the immature hippocampus (38); this leads to structural remodelling of hippocampal CA3 neurons with significant reduction of complexity of apical dendrites and spine density (53–56).

### CRH-Glucocorticoid Interplay

In addition to CRH, glucocorticoids (GC), the end-product of HPA axis, control a distinct HPA-driven regulatory pathway in fetal brain neurogenesis and neural cell proliferation (57, 58). GCs rise over the course of pregnancy to further enhance pCRH release in a distinct pCRH-adrenal GC positive feedback loop (59, 60). This loop also involves placental inflammatory pathways such as RelB and NF-kB2, molecules of the noncanonical NF-kB pathway (61–63). While GCs are key mediators of regulation of fetal growth and maturation of fetal tissues and organs (64, 65), excessive GCs during pregnancy has been associated with adverse fetal outcomes including intrauterine growth restriction (IUGR), cardiovascular disease, metabolic disorders and altered HPA set point of the neonate (18, 66).

Recent studies investigating impact of natural disasters as prenatal stressors associated with abnormal offspring HPA function and development (67), identified raised cortisol as a mediator of an angiogenic phenotype and a crucial role for GCs in altering placental transcriptome, especially reduced expression of GR-regulated endocrine genes expressed in syncytiotrophoblast. Fetal glucocorticoid exposure is partially regulated by the enzyme 11β-hydroxysteroid dehydrogenase type 2 (11β-HSD2), which is abundantly expressed in the placenta and other GC -target tissues catalysing the unidirectional conversion of cortisol to its inactive metabolite cortisone (68), thereby controlling fetal exposure to maternal cortisol. Placental activity and expression levels of 11β-HSD2 have been linked with fetal programming, and down-regulation or deficiency of placental 11β-HSD2 have been associated with unfavourable birth outcomes such as significant restriction in fetal growth and low-birth weight (69, 70). Fetal brain development and limbic brain areas (e.g., amygdala, hippocampus, hypothalamus) are particularly vulnerable to overexposure to high GCs levels. Maternal cortisol levels during pregnancy can predict amygdala volume in childhood and have been associated with temperament of the offspring (71, 72). Moreover, repeated antenatal corticosteroid administration has been linked with lower density of hippocampal neurons of neonates (73), cortical thinning (74) and reduced brain maturation (75) findings that are consistent with animal studies (76–80).

Moreover, a significant rise in GCs levels in response to disease or severe or prolonged stress can impair beneficial effects of CRH on neuronal brain tissue. As numerous studies have linked CRH to neuro-damaging effects in neuronal tissue of prenatally stressed offspring, a key question remains whether CRH is causally linked to structural brain alterations or whether it is an indirect indicator of raised GCs and neurodevelopmental negative outcomes.

## Placental Stress Signals and Fetal Brain Neurotransmitters and GPCRS

In addition to its traditional roles, placenta is recognised as a functional organ supporting fetal central nervous system (CNS) development through adaptive responses to the maternal environment. Recent gene expression and network analysis in murine studies demonstrated that the placenta transcriptome is tightly interconnected with the fetal brain and inhibition of neurotrophin signalling has been identified as a potential mediator of this crosstalk. A pattern of coordinated regulation suggests an extensive network of genes encoding specific receptors and ligands predicted to regulate functional interactions between the placenta and brain (81, 82). Prenatal adverse conditions that activate placenta responses also induce changes in fetal brain neurotransmitter circuits. For example, placental inflammation and raised proinflammatory cytokines (*IL6* and *IL1β*) has been shown to alter the neural expression of dopamine D1 and D2 receptors in brain (83). Other studies have shown that maternal inflammation in midpregnancy results in an upregulation of tryptophan conversion to serotonin (5-HT) within the placenta, leading to exposure of the fetal forebrain to increased concentrations of this biogenic amine and to specific alterations of 5-HT-dependent neurogenic processes (25, 84). 5-HT receptors in the brain are expressed in neurons and glial cells and are involved in many neurodevelopmental events (e.g., neuronal formation, connectivity and synaptic formation) identifying a possible link between the neurodevelopmental complications of the offspring upon changes in the serotonergic system. Altered 5-HT levels can disrupt the expected thalamocortical and intracortical microcircuitry and modify CRH activation *via* the hypothalamic 5-HT1A and 5-HT2A receptors and *via* the 5-HT2C receptors at the hypothalamic paraventricular nucleus (PVN), resulting in HPA axis dysregulation and altered basal activity (21). The γ aminobutyric acid (GABA) system is also sensitive in prenatal environmental insults and the latter can lead to changes in GABAergic gene expression of presynaptic GABAergic genes and GABA receptor. The function of GABA receptors also appears sensitive to such insults: while in the adult brain GABA neurotransmission serves as an inhibitory network, in the fetal and early postnatal brain, GABA signalling is primarily excitatory. Studies revealed that offspring born to immune-challenged mothers, exhibit altered gene expression of genes encoding the two cotransporters involved in the excitatory-to-inhibitory GABA switch, leading to an increased NKCC1:KCC2 ratio and thus experience a delay in the developmental switch of GABA signalling. This might represent a link between early life environmental hits and behavioural changes in adult life (22–24, 50).

## Inflammation and Activation of the HPA Axis

The HPA axis responds to a wide variety of maternal signals that disrupt fetomaternal equilibrium. In particular, maternal immune activation (MIA) during pregnancy by pathogen-derived stimuli, autoinflammatory conditions or environmental irritants (85) might be an important contributor to fetal or early life neurodevelopmental disorders such as spectrum autism disorders and schizophrenia (86–88). For a latest review see Depino A., 2018 (89). The pathophysiological processes implicated in the association between MIA and adverse fetal brain programming, involve not only the hyperactivation of the maternal stress system but also inflammatory processes, elevations in mother’s circulating cytokine levels (e.g. IL-6) and oxidative stress in the maternal and fetal tissues as well as, sometimes, disruption of placental optimal functions due to inflammatory conditions (90, 91). Some prenatal factors that can potentially support fetal resilience to effects of MIA, include high maternal levels of vitamin D, iron and zinc, availability of omega-3 fatty acids and efficient anti-inflammatory and antioxidant response systems. On the other hand, maternal hypoferremia and anaemia, gestational diabetes mellitus, maternal stress during pregnancy, dysbiosis of the maternal gut microbiota and maternal history of cannabinoid exposure (90) can increase the susceptibility of the offspring response to MIA. As inflammatory mediators can pass through fetal blood-brain barrier they can cause neuroinflammation leading to neuronal loss, white matter abnormalities and impaired synaptic development and neurotransmission (92). In addition, studies in rodents suggest that MIA during pregnancy can also result in profound changes in protein synthesis of the fetal brain (involving translation initiation factors and other regulators of protein synthesis) disrupting the neurodevelopment of the fetus (85). MIA due to environmental insults by endocrine disrupting chemicals (EDCS) can also affect the developing embryo by altering synaptic connectivity, neurotransmitter or neuropeptide expression, and neuronal differentiation (93).

MIA appears to induce dysregulation of the bi-directional gut-brain axis (GBA) in particular the gut microbiome and HPA axis. Pro-inflammatory cytokines can modulate the offspring’s HPA axis activity, in particular CRH-R1 expression shown in rodent environmental ‘two-hit’ insult models (94), or modify its neuroimmune function and gut microbial colonization (95). Many studies highlight the role of gut microbiome in health (96) and disease (97). An increasing body of research indicates an association between intestinal microbes and brain function, as intestinal microbes can modulate anxiety-like behaviour and cause endocrine abnormalities in the HPA axis (98). See Morais et al. (99) for a recent review. During pregnancy, maternal microbiome composition, influenced by prenatal conditions (such as MIA), can dynamically affect the offspring and potentially program susceptibility to psychiatric disorders later in life (86, 100, 101). The latter finding is of crucial importance for fetal neurodevelopment as maternal gut microbiome promotes fetal thalamocortical axonogenesis, probably *via* signalling by microbially modulated metabolites to neurons in the developing brain (102). Recent findings suggest a novel interplay between maternal gut microbes and CCL2 in mediating fetal brain inflammation *via* raised IL-6 and placental serotonin metabolism in mediating the programming effects of prenatal stress leading to aberrant sociability and anxiety-like behaviour in adult offspring (25). Moreover, murine studies suggest that early prenatal stress disrupts maternal-to-offspring microbiota transmission and has lasting effects on metabolism, physiology, cognition, and behaviour in male offspring. Maternal vaginal microbiota appears to contribute to the long-lasting effects of prenatal stress on offspring gut and reprogramming of the developing hypothalamus associated with neurodevelopmental disorders (103).

## Epigenetic Mechanisms Linking Stress and Neurodevelopment

Fetal neurodevelopment is associated with considerable epigenetic changes targeting a wide range of genes and molecules with diverse biological roles (104). Recent studies exploring activity of HPA axis in maternal prenatal adversity, demonstrated enhanced methylation of the promoter region of NR3C1, leading to transcription silencing (105, 106). This results in decreased GCs negative feedback and a concomitant increase in both basal and stress-activated (107) HPA activity and elevated CRH and cortisol levels in response to stressors (108, 109). Disruption of key epigenetic processes during critical periods of brain development and the modifications observed in the stress regulatory mechanism, can increase an individual’s vulnerability to psychopathology later in life (110). For example, human post-mortem hippocampal tissue of suicide victims showed reduced GC receptor expression in the hippocampus of those with a history of childhood abuse (111). In previous studies, NR3C1 methylation has also been associated with internalizing psychopathology in children and adolescents (112, 113). Other perceived stressors, such as socioeconomic status, health conditions, and lifestyle can also influence NR3C1 gene regulation, revealing the complexity of environmental impacts on epigenetic modifications (114). During human hippocampal neurogenesis, exposure to GCs can lead to lasting DNA methylation changes and subsequent alterations in gene transcription (115). Similar findings have been described in guinea pigs with the intriguing finding that hippocampal changes in gene transcription and DNA methylation persist across three generations of the juvenile female offspring (116).

The impact of stress-induced methylation on CRH gene expression is not well understood. Recent studies identified positive association between prenatal maternal stress and methylation of the transcription factor binding site of CRH gene in the neonatal cord blood, maternal and placenta blood samples. Methylation in several *NR3C1* and *CRH* CpG sites, predicted a negative correlation with birth weight (117). Maternal pCRH concentration correlates with cord blood cells DNA methylation, especially methylation of the leptin (*LEP*) gene promoter, and these epigenetic alterations are present into mid-childhood. As higher LEP methylation has been associated with lower BMI in childhood, these results might suggest an underlying link between pCRH and metabolic fetal programming (118). Studies on rodents reveal that chronic stress can induce epigenetic alterations that can mould the central stress response and ultimately affect gene expression and CRH transcriptional and translational activities in many brain areas, in a sex specific manner (119). Although maternal interaction is a major determinant of hypothalamic CRH expression in early life (28), data from *in utero* epigenetic studies are not available but required to explore the impact of prenatal interaction on later-life stress responsiveness. DNA methylation is not the only epigenetic alteration that can be detected; early life stress can also lead to histone modifications, that can modify chromatin architecture, alter transcription, and ultimately affect gene expression of candidate genes during early brain development (120–122). Chronic maternal stress can also generate major alterations in the antioxidant levels, and in the cellular pathways implicated in neurodevelopmental processes and DNA damage; a recent study demonstrated that maternal chronic stress downregulates levels of β-catenin and BDNF and upregulates GSK-3β, resulting in compromised neurogenesis in the prenatally stressed offspring (41, 123). Long-term alterations on signalling pathways interfering with the inflammatory/immune response and metabolism in the prenatally stressed offspring, have been reported especially with the neuroinflammation signalling pathway, the NF-kB and p38 MAPK signalling (124). Epigenetic changes may also interfere with GPCR signalling and changes in the methylation status of genes encoding G proteins or GPCRs may result in inability of G protein signalling initiation or even in abruption of the GPCR signalling transduction. For example, in male neonates, there is a positive correlation between pregnancy anxiety and fetal methylation of the *GABBR1* gene, that encodes the G protein coupled receptor subunit GABA-B1 (26). Likewise, maternal emotional stress and cortisol levels during pregnancy are associated with fetal DNA methylation of GNASXL, the extra large isoform of Gas protein involved in networks that control fetal growth and development (125). For a recent review see Cao-Lei et al. (126).

## Conclusions

**Figure 1:**
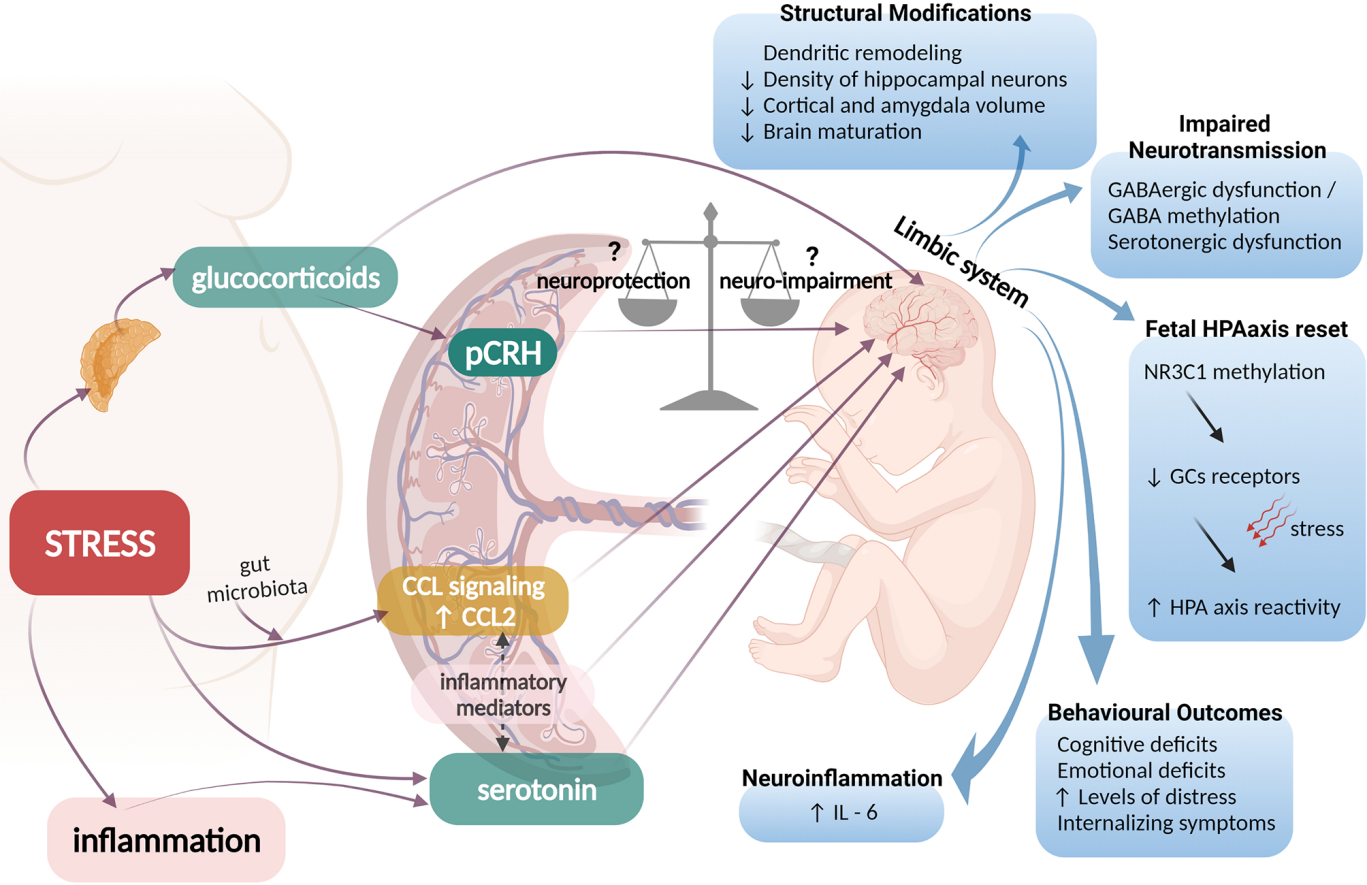
Possible pathophysiological mechanisms linking prenatal maternal adversity to disrupted fetal brain programming. Maternal stress could activate adrenal production of glucocorticoids (GCs) that can cross the placenta and regulate fetal brain neurogenesis. GCs also enhance production and release of placental CRH (pCRH) into the fetal compartments, a neuropeptide that can exert either neuroprotective or neuro-impairment effects. Excess levels of GCs and pCRH have been associated with structural fetal brain modifications, impaired neurotransmission and disrupted programming of the HPA axis of the fetus that involves epigenetic modifications of the glucocorticoid receptor (GR) gene and is linked with increased HPA axis reactivity of the neonate and adverse behavioral and emotional outcomes later in life. Additionally, maternal stress or inflammatory conditions can enhance placental output of serotonin (5-HT) to the fetal brain leading to serotonergic dysfunction. Excess maternal stress, can influence signals arising from gut microbiota to affect placental CCL signaling. The interplay between placental 5-HT, CCL-2 and other inflammatory mediators ultimately drives fetal neuroinflammation and IL-6 elevation in the fetal brain.

The placenta is pivotal in the development of the fetal brain and extensive molecular networks and pathways functionally link the two tissues. Placental adaptive inflammation and epigenetic responses to the maternal environment under the influence of prenatal stress, activate mechanisms that exert adverse roles in fetal neurodevelopment (**Figure 1**). The underlying biological mechanisms only recently began to unravel, especially the roles of altered HPA responses involving placental CRH and glucocorticoids. However, the precise mechanisms employed by the fetus to protect itself from an unfavourable intrauterine environment have not yet been fully elucidated. The fetus plays an active role in its own development and efforts to establish successful pregnancy outcomes, and *via* developmental programming, alters the birth phenotype to adjust better to the postnatal life. This “fight response”, is controlled by the HPA at multiple levels with pCRH exerting a major influence by integrating the homeostatic mechanisms that will ultimately promote adaptation to maternal adversity. Nonetheless, in cases of extremely unfavourable intrauterine conditions, the fetus can also choose to ‘escape’ from a hostile maternal environment, triggering the “flight response” *via* HPA axis activation, that will ultimately initiate parturition and lead to preterm birth. This hypothesis places pCRH in a central role in controlling the placental “clock” determining the length of pregnancy and the onset of labour (32, 39, 127–129) but at extreme situations at the expense of optimal fetal development. The impact of HPA and pCRH activation on neurodevelopment is crucial and dissecting these actions could provide novel mechanistic (and potentially actionable) insights especially for understanding susceptibility to psychosomatic disorders later in life.

## Author Contributions

All authors contributed to the article and approved the submitted version.

## Conflict of Interest

The authors declare that the research was conducted in the absence of any commercial or financial relationships that could be construed as a potential conflict of interest.

## Publisher’s Note

All claims expressed in this article are solely those of the authors and do not necessarily represent those of their affiliated organizations, or those of the publisher, the editors and the reviewers. Any product that may be evaluated in this article, or claim that may be made by its manufacturer, is not guaranteed or endorsed by the publisher.

## References

[1] First 1000 days of life. House Commons, Heal Soc Care Comm. In: 13th Report: Session 2017-2019 (2019). Available at: www.parliament.uk/copyright.

[2] Russell JA Brunton PJ. Giving a Good Start to a New Life via Maternal Brain Allostatic Adaptations in Pregnancy. Front Neuroendocrinol (2019) 53:100739. 10.1016/j.yfrne.2019.02.003 30802468

[3] Ulupınar E. Effects of Prenatal Stress on Developmental Anatomy of the Brain and Adult Behavioural Pathology. Anat (Int J Exp Clin Anat) (2009) 3:3–13. 10.2399/ana.09.034

[4] Pervanidou P Chrousos GP. Early-Life Stress: From Neuroendocrine Mechanisms to Stress-Related Disorders. Horm Res Paediatr (2018) 89:372–9. 10.1159/000488468 29886495

[5] Padmanabhan V Cardoso RC Puttabyatappa M. Developmental Programming, a Pathway to Disease. Endocrinology (2016) 157:1328–40. 10.1210/en.2016-1003 PMC4816734 26859334

[6] Entringer S Kumsta R Nelson EL Hellhammer DH Wadhwa PD Wüst S. Influence of Prenatal Psychosocial Stress on Cytokine Production in Adult Women. Dev Psychobiol (2008) 50:579–87. 10.1002/dev.20316 PMC2838479 18683180

[7] Veru F Laplante DP Luheshi G King S. Prenatal Maternal Stress Exposure and Immune Function in the Offspring. Int J Biol Stress (2014) 17:133–48. 10.3109/10253890.2013.876404 24417382

[8] Li J Olsen J Vestergaard M Obel C Baker JL Sørensen TIA. Prenatal Stress Exposure Related to Maternal Bereavement and Risk of Childhood Overweight. PloS One (2010) 5:e11896. 10.1371/journal.pone.0011896 20689593 PMC2912844

[9] Entringer S Wüst S Kumsta R Layes IM Nelson EL Hellhammer DH. Prenatal Psychosocial Stress Exposure Is Associated With Insulin Resistance in Young Adults. Am J Obstet Gynecol (2008) 199:498.e1–7. 10.1016/j.ajog.2008.03.006 PMC3587039 18448080

[10] Valsamakis G Papatheodorou D Chalarakis N Manolikaki M Margeli A Papassotiriou I. Maternal Chronic Stress Correlates With Serum Levels of Cortisol, Glucose and C-Peptide in the Fetus, and Maternal Non Chronic Stress With Fetal Growth. Psychoneuroendocrinology (2020) 114:104591. 10.1016/j.psyneuen.2020.104591 32007670

[11] Brunton PJ. Neuroactive Steroids and Stress Axis Regulation: Pregnancy and Beyond. J Steroid Biochem Mol Biol (2016) 160:160–8. 10.1016/j.jsbmb.2015.08.003 26259885

[12] Van Den Bergh BRH Van Calster B Smits T Van Huffel S Lagae L. Antenatal Maternal Anxiety Is Related to HPA-Axis Dysregulation and Self-Reported Depressive Symptoms in Adolescence: A Prospective Study on the Fetal Origins of Depressed Mood. Neuropsychopharmacology (2008) 33:536–45. 10.1038/sj.npp.1301450 17507916

[13] O’Connor TG Heron J Golding J Beveridge M Glover V. Maternal Antenatal Anxiety and Children’s Behavioural/Emotional Problems at 4 Years. Report From the Avon Longitudinal Study of Parents and Children. Br J Psychiatry (2002) 180:502–8. 10.1192/bjp.180.6.502 12042228

[14] Barker ED Jaffee SR Uher R Maughan B. The Contribution of Prenatal and Postnatal Maternal Anxiety and Depression to Child Maladjustment. Depress Anxiety (2011) 28:696–702. 10.1002/da.20856 21769997

[15] Li J Olsen J Vestergaard M Obel C. Attention-Deficit/Hyperactivity Disorder in the Offspring Following Prenatal Maternal Bereavement: A Nationwide Follow-Up Study in Denmark. Eur Child Adolesc Psychiatry (2010) 19:747–53. 10.1007/s00787-010-0113-9 20495989

[16] Howland MA Sandman CA Glynn LM Crippen C Davis EP. Fetal Exposure to Placental Corticotropin-Releasing Hormone Is Associated With Child Self-Reported Internalizing Symptoms. Psychoneuroendocrinology (2016) 67:10–7. 10.1016/j.psyneuen.2016.01.023 PMC4808336 26855003

[17] Tau GZ Peterson BS. Normal Development of Brain Circuits. Neuropsychopharmacology (2010) 35:147–68. 10.1038/npp.2009.115 PMC3055433 19794405

[18] Baud O Berkane N. Hormonal Changes Associated With Intra-Uterine Growth Restriction: Impact on the Developing Brain and Future Neurodevelopment. Front Endocrinol (Lausanne) (2019) 10:179. 10.3389/fendo.2019.00179 30972026 PMC6443724

[19] Langenhan T Piao X Monk KR. Adhesion G Protein-Coupled Receptors in Nervous System Development and Disease. Nat Rev Neurosci (2016) 17:550–61. 10.1038/nrn.2016.86 27466150

[20] McCorvy JD Roth BL. Structure and Function of Serotonin G Protein-Coupled Receptors. Pharmacol Ther (2015) 150:129–42. 10.1016/j.pharmthera.2015.01.009 PMC4414735 25601315

[21] Hanswijk SI Spoelder M Shan L Verheij MMM Muilwijk OG Li W. Gestational Factors Throughout Fetal Neurodevelopment: The Serotonin Link. Int J Mol Sci (2020) 21:5850. 10.3390/ijms21165850 PMC7461571 32824000

[22] Richetto J Calabrese F Riva MA Meyer U. Prenatal Immune Activation Induces Maturation-Dependent Alterations in the Prefrontal GABAergic Transcriptome. Schizophr Bull (2014) 40:351–61. 10.1093/schbul/sbs195 PMC3932076 23328159

[23] Pozzi D Rasile M Corradini I Matteoli M. Environmental Regulation of the Chloride Transporter KCC2: Switching Inflammation Off to Switch the GABA on? Transl Psychiatry (2020) 10:349. 10.1038/s41398-020-01027-6 33060559 PMC7562743

[24] Nyffeler M Meyer U Yee BK Feldon J Knuesel I. Maternal Immune Activation During Pregnancy Increases Limbic GABAA Receptor Immunoreactivity in the Adult Offspring: Implications for Schizophrenia. Neuroscience (2006) 143:51–62. 10.1016/j.neuroscience.2006.07.029 17045750

[25] Chen HJ Antonson AM Rajasekera TA Patterson JM Bailey MT Gur TL. Prenatal Stress Causes Intrauterine Inflammation and Serotonergic Dysfunction, and Long-Term Behavioral Deficits Through Microbe- and CCL2-Dependent Mechanisms. Transl Psychiatry (2020) 10:191. 10.1038/s41398-020-00876-5 32546752 PMC7297973

[26] Vangeel EB Pishva E Hompes T van den Hove D Lambrechts D Allegaert K. Newborn Genome-Wide DNA Methylation in Association With Pregnancy Anxiety Reveals a Potential Role for GABBR1. Clin Epigenet (2017) 9:107. 10.1186/s13148-017-0408-5 PMC5627482 29026448

[27] Bateson P Barker D Clutton-Brock T Deb D D’Udine B Foley RA. Developmental Plasticity and Human Health. Nature (2004) 430:419–21. 10.1038/nature02725 15269759

[28] Howland MA Sandman CA Glynn LM. Developmental Origins of the Human Hypothalamic-Pituitary-Adrenal Axis. Expert Rev Endocrinol Metab (2017) 12:321–39. 10.1080/17446651.2017.1356222 PMC6334849 30058893

[29] Mastorakos G Ilias I. Maternal Hypothalamic – Pituitary – Adrenal Axis in Pregnancy and the Postpartum Period. Ann NY Acad Sci (2006) 900:95–106. 10.1111/j.1749-6632.2000.tb06220.x 10818396

[30] Riley SC Walton JC Herlick JM Challis JRG. The Localization and Distribution of Corticotropin-Releasing Hormone in the Human Placenta and Fetal Membranes Throughout Gestation. J Clin Endocrinol Metab (1991) 72:1001–7. 10.1210/jcem-72-5-1001 2022703

[31] Lovejoy DA Chang B Lovejoy N Castillo J. Molecular Evolution of GPCRs: CRH/CRH Receptors. J Mol Endocrinol (2014) 52:43–60. 10.1530/JME-13-0238 24711645

[32] Alcántara-Alonso V Panetta P de Gortari P Grammatopoulos DK. Corticotropin-Releasing Hormone as the Homeostatic Rheostat of Feto-Maternal Symbiosis and Developmental Programming In Utero and Neonatal Life. Front Endocrinol (Lausanne) (2017) 8:161. 10.3389/fendo.2017.00161 28744256 PMC5504167

[33] Leviton A Allred EN Kuban KCK O’Shea TM Paneth N Majzoub J. Brain Disorders Associated With Corticotropin-Releasing Hormone Expression in the Placenta Among Children Born Before the 28th Week of Gestation. Acta Paediatr Int J Paediatr (2016) 105:7–11. 10.1111/apa.13174 PMC4701600 26331704

[34] Koutmani Y Politis PK Elkouris M Agrogiannis G Kemerli M Patsouris E. Corticotropin-Releasing Hormone Exerts Direct Effects on Neuronal Progenitor Cells: Implications for Neuroprotection. Mol Psychiatry (2013) 18:300–7. 10.1038/mp.2012.198 PMC3578949 23380766

[35] Koutmani Y Gampierakis IA Polissidis A Ximerakis M Koutsoudaki PN Polyzos A. CRH Promotes the Neurogenic Activity of Neural Stem Cells in the Adult Hippocampus. Cell Rep (2019) 29:932–45. 10.1016/j.celrep.2019.09.037 31644914

[36] Sandman CA Curran MM Davis EP Glynn LM Head K Baram TZ. Cortical Thinning and Neuropsychiatric Outcomes in Children Exposed to Prenatal Adversity: A Role for Placental CRH? Am J Psychiatry (2018) 175:471–9. 10.1176/appi.ajp.2017.16121433 PMC5930042 29495899

[37] Curran MM Sandman CA Davis EP Glynn LM Baram TZ. Abnormal Dendritic Maturation of Developing Cortical Neurons Exposed to Corticotropin Releasing Hormone (CRH): Insights Into Effects of Prenatal Adversity? PloS One (2017) 12:1–11. 10.1371/journal.pone.0180311 PMC5489219 28658297

[38] Chen Y Bender RA Brunson KL Pomper JK Grigoriadis DE Wurst W. Modulation of Dendritic Differentiation by Corticotropin-Releasing Factor in the Developing Hippocampus. Proc Natl Acad Sci USA (2004) 101:15782–7. 10.1073/pnas.0403975101 PMC524840 15496472

[39] Sandman CA. Fetal Exposure to Placental Corticotropin-Releasing Hormone (pCRH) Programs Developmental Trajectories. Peptides (2015) 72:145–53. 10.1016/j.peptides.2015.03.020 PMC4777695 25841879

[40] Bayatti N Zschocke J Behl C. Brain Region-Specific Neuroprotective Action and Signaling of Corticotropin-Releasing Hormone in Primary Neurons. Endocrinology (2003) 144:4051–60. 10.1210/en.2003-0168 12933679

[41] Fatima M Srivastav S Ahmad MH Mondal AC. Effects of Chronic Unpredictable Mild Stress Induced Prenatal Stress on Neurodevelopment of Neonates: Role of GSK-3β. Sci Rep (2019) 9:1305. 10.1038/s41598-018-38085-2 30718708 PMC6361942

[42] Jeanneteau FD Lambert WM Ismaili N Bath KG Lee FS Garabedian MJ. BDNF and Glucocorticoids Regulate Corticotrophin-Releasing Hormone (CRH) Homeostasis in the Hypothalamus. Proc Natl Acad Sci USA (2012) 109:1305–10. 10.1073/pnas.1114122109 PMC3268297 22232675

[43] Hanstein R Lu A Wurst W Holsboer F Deussing JM Clement AB. Transgenic Overexpression of Corticotropin Releasing Hormone Provides Partial Protection Against Neurodegeneration in an In Vivo Model of Acute Excitotoxic Stress. Neuroscience (2008) 156:712–21. 10.1016/j.neuroscience.2008.07.034 18708129

[44] Boersma GJ Lee RS Cordner ZA Ewald ER Purcell RH Moghadam AA. Prenatal Stress Decreases Bdnf Expression and Increases Methylation of Bdnf Exon IV in Rats. Epigenetics (2014) 9:437–47. 10.4161/epi.27558 PMC4053462 24365909

[45] Blaze J Asok A Borrelli K Tulbert C Bollinger J Ronca AE. Intrauterine Exposure to Maternal Stress Alters Bdnf IV DNA Methylation and Telomere Length in the Brain of Adult Rat Offspring. Int J Dev Neurosci (2017) 62:56–62. 10.1016/j.ijdevneu.2017.03.007 28330827 PMC5600826

[46] Lautarescu A Craig MC Glover V. Prenatal Stress: Effects on fetal and Child Brain Development. Clin Epigenet (2020) 150:17–40. 10.1016/bs.irn.2019.11.002 32204831

[47] Lautarescu A Craig MC Glover V. Prenatal Stress: Effects on Fetal and Child Brain Development. Inter Rev Neurobiol (2020) 150:17–40. 10.1016/bs.irn.2019.11.002 32204831

[48] Gray JD Kogan JF Marrocco J McEwen BS. Genomic and Epigenomic Mechanisms of Glucocorticoids in the Brain. Nat Rev Endocrinol (2017) 13:661–73. 10.1038/nrendo.2017.97 28862266

[49] Davis EP Glynn LM Schetter CD Hobel C Chicz-Demet A Sandman CA. Corticotropin-Releasing Hormone During Pregnancy Is Associated With Infant Temperament. Dev Neurosci (2005) 27:299–305. 10.1159/000086709 16137987

[50] Shang Y Chen R Li F Zhang H Wang H Zhang T. Prenatal Stress Impairs Memory Function in the Early Development of Male- Offspring Associated With the Gaba Function. Physiol Behav (2021) 228:113184. 10.1016/j.physbeh.2020.113184 32979340

[51] Hermes M Antonow-schlorke I Hollstein D Kuehnel S Rakers F Frauendorf V. Maternal Psychosocial Stress During Early Gestation Impairs Fetal Structural Brain Development in Sheep. Stress (2020) 23:233–42. 10.1080/10253890.2019.1652266 31469022

[52] Wu Y Lu Y Jacobs M Pradhan S Kapse K Zhao L. Association of Prenatal Maternal Psychological Distress With Fetal Brain Growth, Metabolism, and Cortical Maturation. JAMA Netw Open (2020) 3(1):e1919940. 10.1001/jamanetworkopen.2019.19940 31995213 PMC6991285

[53] Wang XD Chen Y Wolf M Wagner KV Liebl C Scharf SH. Forebrain CRHR1 Deficiency Attenuates Chronic Stress-Induced Cognitive Deficits and Dendritic Remodeling. Neurobiol Dis (2011) 42:300–10. 10.1016/j.nbd.2011.01.020 PMC3200197 21296667

[54] Liu R Yang XD Liao XM Xie XM Su YA Li JT. Early Postnatal Stress Suppresses the Developmental Trajectory of Hippocampal Pyramidal Neurons: The Role of CRHR1. Brain Struct Funct (2016) 221:4525–36. 10.1007/s00429-016-1182-4 26792004

[55] Liao XM Yang XD Jia J Li JT Xie XM Su YA. Blockade of Corticotropin-Releasing Hormone Receptor 1 Attenuates Early-Life Stress-Induced Synaptic Abnormalities in the Neonatal Hippocampus. Hippocampus (2014) 24:528–40. 10.1002/hipo.22254 24493406

[56] Ivy AS Rex CS Chen Y Dubé C Maras PM Grigoriadis DE. Hippocampal Dysfunction and Cognitive Impairments Provoked by Chronic Early-Life Stress Involve Excessive Activation of CRH Receptors. J Neurosci (2010) 30:13005–15. 10.1523/JNEUROSCI.1784-10.2010 PMC2991143 20881118

[57] Anacker C Cattaneo A Luoni A Musaelyan K Zunszain PA Milanesi E. Glucocorticoid-Related Molecular Signaling Pathways Regulating Hippocampal Neurogenesis. Neuropsychopharmacology (2013) 38:872–83. 10.1038/npp.2012.253 PMC3672002 23303060

[58] Odaka H Adachi N Numakawa T. Impact of Glucocorticoid on Neurogenesis. Neural Regener Res (2017) 12:1028–35. 10.4103/1673-5374.211174 PMC5558474 28852377

[59] Nicolaides NC Kyratzi E Lamprokostopoulou A Chrousos GP Charmandari E. Stress, the Stress System and the Role of Glucocorticoids. Neuroimmunomodulation (2014) 22:6–19. 10.1159/000362736 25227402

[60] Solano ME Arck PC. Steroids, Pregnancy and Fetal Development. Front Immunol (2020) 10:3017. 10.3389/fimmu.2019.03017 32038609 PMC6987319

[61] Wang B Parobchak N Rosen T. RelB/NF-κB2 Regulates Corticotropin-Releasing Hormone in the Human Placenta. Mol Endocrinol (2012) 26:1356–69. 10.1210/me.2012-1035 PMC5416986 22734038

[62] Wang B Palomares K Parobchak N Cece J Rosen M Nguyen A. Glucocorticoid Receptor Signaling Contributes to Constitutive Activation of the Noncanonical NF-κB Pathway in Term Human Placenta. Mol Endocrinol (2013) 27:203–11. 10.1210/me.2012-1309 PMC5417329 23239753

[63] Di Stefano V Wang B Parobchak N Roche N Rosen T. RelB/p52-Mediated NF-kB Signaling Alters Histone Acetylation to Increase the Abundance of Corticotropin-Releasing Hormone in Human Placenta. Sci Signal (2015) 8:ra85. 10.1126/scisignal.aaa9806 26307012

[64] Rog-Zielinska EA Richardson RV Denvir MA Chapman KE. Glucocorticoids and Foetal Heart Maturation; Implications for Prematurity and Foetal Programming. J Mol Endocrinol (2014) 52:125–35. 10.1530/JME-13-0204 24299741

[65] McGoldrick E Brown J Middleton P McKinlay CJ Haas DM Crowther CA. Antenatal Corticosteroids for Fetal Lung Maturation: An Overview of Cochrane Reviews. Cochrane Database Syst Rev (2016) 2016(4):CD012156. 10.1002/14651858.CD012156

[66] O’Donnell KJ Meaney MJ. Fetal Origins of Mental Health: The Developmental Origins of Health and Disease Hypothesis. Am J Psychiatry (2017) 174:319–28. 10.1176/appi.ajp.2016.16020138 27838934

[67] Nomura Y Rompala G Pritchett L Aushev V Chen J Hurd YL. Natural Disaster Stress During Pregnancy Is Linked to Reprogramming of the Placenta Transcriptome in Relation to Anxiety and Stress Hormones in Young Offspring. Mol Psychiatry (2021). 10.1038/s41380-021-01123-z PMC8586067 33981007

[68] Niu P Yang K. The 11β-Hydroxysteroid Dehydrogenase Type 2 Activity in Human Placental Microsomes Is Inactivated by Zinc and the Sulfhydryl Modifying Reagent N-Ethylmaleimide. Biochim Biophys Acta - Protein Struct Mol Enzymol (2002) 1594:364–71. 10.1016/S0167-4838(01)00329-6 11904232

[69] Konstantakou P Mastorakos G Vrachnis N Tomlinson JW Valsamakis G. Dysregulation of 11beta-Hydroxysteroid Dehydrogenases: Implications During Pregnancy and Beyond. J Matern Neonatal Med (2017) 30:284–93. 10.3109/14767058.2016.1171308 27018008

[70] Cottrell EC Seckl JR Holmes MC Wyrwoll CS. Foetal and Placental 11β-HSD2: A Hub for Developmental Programming. Acta Physiol (2014) 210:288–95. 10.1111/apha.12187 24330050

[71] Buss C Davis EP Shahbaba B Pruessner JC Head K Sandman CA. Maternal Cortisol Over the Course of Pregnancy and Subsequent Child Amygdala and Hippocampus Volumes and Affective Problems. Proc Natl Acad Sci USA (2012) 109:1312–9. 10.1073/pnas.1201295109 PMC3356611 22529357

[72] Davis EP Glynn LM Schetter CD Hobel C Chicz-Demet A Sandman CA. Prenatal Exposure to Maternal Depression and Cortisol Influences Infant Temperament. J Am Acad Child Adolesc Psychiatry (2007) 46:737–46. 10.1097/chi.0b013e318047b775 17513986

[73] Tijsseling D Wijnberger LDE Derks JB van Velthoven CTJ de Vries WB van Bel F. Effects of Antenatal Glucocorticoid Therapy on Hippocampal Histology of Preterm Infants. PloS One (2012) 7:e33369. 10.1371/journal.pone.0033369 22457757 PMC3311632

[74] Davis EP Sandman CA Buss C Wing DA Head K. Fetal Glucocorticoid Exposure Is Associated With Preadolescent Brain Development. Biol Psychiatry (2013) 74:647–55. 10.1016/j.biopsych.2013.03.009 PMC3985475 23611262

[75] Modi N Lewis H Al-Naqeeb N Ajayi-Obe M Dore CJ Rutherford M. The Effects of Repeated Antenatal Glucocorticoid Therapy on the Developing Brain. Pediatr Res (2001) 50:581–5. 10.1203/00006450-200111000-00008 11641451

[76] Kanagawa T Tomimatsu T Hayashi S Shioji M Fukuda H Shimoya K. The Effects of Repeated Corticosteroid Administration on the Neurogenesis in the Neonatal Rat. Am J Obstet Gynecol (2006) 194:231–8. 10.1016/j.ajog.2005.06.015 16389037

[77] Huang WL Beazley LD Quinlivan JA Evans SF Newnham JP Dunlop SA. Effect of Corticosteroids on Brain Growth in Fetal Sheep. Obstet Gynecol (1999) 94:213–8. 10.1016/S0029-7844(99)00265-3 10432130

[78] Noorlander CW Visser GH Ramakers GM Nikkels PG de GP. Prenatal Corticosteroid Exposure Affects Hippocampal Plasticity and Reduces Lifespan. Dev Neurobiol (2008) 68:237–46. 10.1002/dneu 18000831

[79] Constantinof A Moisiadis VG Kostaki A Szyf M Matthews SG. Antenatal Glucocorticoid Exposure Results in Sex-Specific and Transgenerational Changes in Prefrontal Cortex Gene Transcription That Relate to Behavioural Outcomes. Sci Rep (2019) 9:764. 10.1038/s41598-018-37088-3 30679753 PMC6346022

[80] Moisiadis VG Mouratidis A Kostaki A Matthews SG. A Single Course of Synthetic Glucocorticoids in Pregnant Guinea Pigs Programs Behavior and Stress Response in Two Generations of Offspring. Endocrinology (2018) 159:4065–76. 10.1210/en.2018-00666 PMC6262222 30383219

[81] Behura SK Dhakal P Kelleher AM Balboula A Patterson A Spencer TE. The Brain-Placental Axis: Therapeutic and Pharmacological Relevancy to Pregnancy. Pharmacol Res (2019) 149:104468. 10.1016/j.phrs.2019.104468 31600597 PMC6944055

[82] Behura SK Kelleher AM Spencer TE. Evidence for Functional Interactions Between the Placenta and Brain in Pregnant Mice. FASEB J (2019) 33:4261–72. 10.1096/fj.201802037R PMC6404589 30521381

[83] Bronson SL Bale TL. Prenatal Stress-Induced Increases in Placental Inflammation and Offspring Hyperactivity Are Male-Specific and Ameliorated by Maternal Antiinflammatory Treatment. Endocrinology (2014) 155:2635–46. 10.1210/en.2014-1040 PMC4060181 24797632

[84] Goeden N Velasquez J Arnold KA Chan Y Lund BT Anderson GM. Maternal Inflammation Disrupts Fetal Neurodevelopment via Increased Placental Output of Serotonin to the Fetal Brain. J Neurosci (2016) 36:6041–9. 10.1523/JNEUROSCI.2534-15.2016 PMC4887568 27251625

[85] Zengeler KE Lukens JR. Inflammation Stresses Out Brain Development. Nat Neurosci (2021) 24:155–7. 10.1038/s41593-020-00775-4 33361821

[86] Kim S Kim H Yim YS Ha S Atarashi K Tan TG. Maternal Gut Bacteria Promote Neurodevelopmental Abnormalities in Mouse Offspring. Nature (2017) 549:528–32. 10.1038/nature23910 PMC5870873 28902840

[87] Brown AS. Epidemiologic Studies of Exposure to Prenatal Infection and Risk of Schizophrenia and Autism. Dev Neurobiol (2012) 72:1272–6. 10.1002/dneu.22024 PMC3435457 22488761

[88] Elovitz MA Brown AG Breen K Anton L Maubert M Burd I. Intrauterine Inflammation, Insufficient to Induce Parturition, Still Evokes Fetal and Neonatal Brain Injury. Int J Dev Neurosci (2011) 29:663–71. 10.1016/j.ijdevneu.2011.02.011 PMC3140629 21382466

[89] Depino AM. Perinatal Inflammation and Adult Psychopathology: From Preclinical Models to Humans. Semin Cell Dev Biol (2018) 77:104–14. 10.1016/j.semcdb.2017.09.010 28890420

[90] Meyer U. Neurodevelopmental Resilience and Susceptibility to Maternal Immune Activation. Trends Neurosci (2019) 42:793–806. 10.1016/j.tins.2019.08.001 31493924

[91] Bergdolt L Dunaevsky A. Brain Changes in a Maternal Immune Activation Model of Neurodevelopmental Brain Disorders. Prog Neurobiol (2019) 175:1–19. 10.1016/j.pneurobio.2018.12.002 30590095 PMC6413503

[92] Knuesel I Chicha L Britschgi M Schobel SA Bodmer M Hellings JA. Maternal Immune Activation and Abnormal Brain Development Across CNS Disorders. Nat Rev Neurol (2014) 10:643–60. 10.1038/nrneurol.2014.187 25311587

[93] Nesan D Kurrasch DM. Gestational Exposure to Common Endocrine Disrupting Chemicals and Their Impact on Neurodevelopment and Behavior. Annu Rev of Physiol (2020) 82:177–202. 10.1146/annurev-physiol-021119-034555 31738670

[94] Katz-Barber MW Hollins SL Cuskelly A Leong AJW Dunn A Harms L. Investigating the Gut-Brain Axis in a Neurodevelopmental Rodent Model of Schizophrenia. Brain Behav Immun - Heal (2020) 3:100048. 10.1016/j.bbih.2020.100048 PMC8474551 34589838

[95] Jiang NM Cowan M Moonah SN Petri WA. The Impact of Systemic Inflammation on Neurodevelopment. Trends Mol Med (2018) 24:794–804. 10.1016/j.molmed.2018.06.008 30006148 PMC6110951

[96] Valdes AM Walter J Segal E Spector TD. Role of the Gut Microbiota in Nutrition and Health. BMJ (2018) 361:k2179. 10.1136/bmj.k2179 29899036 PMC6000740

[97] Moossavi S Bishehsari F. Microbes: Possible Link Between Modern Lifestyle Transition and the Rise of Metabolic Syndrome. Obes Rev (2019) 20:407–19. 10.1111/obr.12784 30548384

[98] Huo R Zeng B Zeng L Cheng K Li B Luo Y. Microbiota Modulate Anxiety-Like Behavior and Endocrine Abnormalities in Hypothalamic-Pituitary-Adrenal Axis. Front Cell Infect Microbiol (2017) 7:489. 10.3389/fcimb.2017.00489 29250490 PMC5715198

[99] Morais LH Schreiber HL Mazmanian SK. The Gut Microbiota–Brain Axis in Behaviour and Brain Disorders. Nat Rev Microbiol (2021) 19:241–55. 10.1038/s41579-020-00460-0 33093662

[100] Codagnone MG Spichak S O’Mahony SM O’Leary OF Clarke G Stanton C. Programming Bugs: Microbiota and the Developmental Origins of Brain Health and Disease. Biol Psychiatry (2019) 85:150–63. 10.1016/j.biopsych.2018.06.014 30064690

[101] Lammert CR Frost EL Bolte AC Paysour MJ Shaw ME Bellinger CE. Cutting Edge: Critical Roles for Microbiota-Mediated Regulation of the Immune System in a Prenatal Immune Activation Model of Autism. J Immunol (2018) 201:845–50. 10.4049/jimmunol.1701755 PMC6057827 29967099

[102] Vuong HE Pronovost GN Williams DW Coley EJL Siegler EL Qiu A. The Maternal Microbiome Modulates Fetal Neurodevelopment in Mice. Nature (2020) 586:281–6. 10.1038/s41586-020-2745-3 PMC7554197 32968276

[103] Jašarević E Howard CD Morrison K Misic A Weinkopff T Scott P. The Maternal Vaginal Microbiome Partially Mediates the Effects of Prenatal Stress on Offspring Gut and Hypothalamus. Nat Neurosci (2018) 21:1061–71. 10.1038/s41593-018-0182-5 29988069

[104] Spiers H Hannon E Schalkwyk LC Smith R Wong CCY O’Donovan MC. Methylomic Trajectories Across Human Fetal Brain Development. Genome Res (2015) 25:338–52. 10.1101/gr.180273.114 PMC4352878 25650246

[105] Liu PZ Nusslock R. How Stress Gets Under the Skin: Early Life Adversity and Glucocorticoid Receptor Epigenetic Regulation. Curr Genomics (2018) 19:653–64. 10.2174/1389202919666171228164350 PMC6225447 30532645

[106] Daskalakis NP Yehuda R. Site-Specific Methylation Changes in the Glucocorticoid Receptor Exon 1F Promoter in Relation to Life Adversity: Systematic Review of Contributing Factors. Front Neurosci (2014) 8:369. 10.3389/fnins.2014.00369 25484853 PMC4240065

[107] Bleker LS De Rooij SR Roseboom TJ. Programming Effects of Prenatal Stress on Neurodevelopment—The Pitfall of Introducing a Self- Fulfilling Prophecy. Int J Environ Res Public Health (2019) 16:2301. 10.3390/ijerph16132301 PMC6651796 31261808

[108] Heim C Nemeroff CB. The Role of Childhood Trauma in the Neurobiology of Mood and Anxiety Disorders: Preclinical and Clinical Studies. Biol Psychiatry (2001) 49:1023–39. 10.1016/S0006-3223(01)01157-X 11430844

[109] Oberlander TF Weinberg J Papsdorf M Grunau R Misri S Devlin AM. Prenatal Exposure to Maternal Depression, Neonatal Methylation of Human Glucocorticoid Receptor Gene (NR3C1) and Infant Cortisol Stress Responses. Epigenetics (2008) 3:97–106. 10.4161/epi.3.2.6034 18536531

[110] McGowan PO Matthews SG. Prenatal Stress, Glucocorticoids, and Developmental Programming of the Stress Response. Endocrinology (2018) 159:69–82. 10.1210/en.2017-00896 29136116

[111] McGowan PO Sasaki A D’Alessio AC Dymov S Labonté B Szyf M. Epigenetic Regulation of the Glucocorticoid Receptor in Human Brain Associates With Childhood Abuse. Nat Neurosci (2009) 12:342–8. 10.1038/nn.2270 PMC2944040 19234457

[112] Efstathopoulos P Andersson F Melas PA Yang LL Villaescusa JC Ruegg J. NR3C1 Hypermethylation in Depressed and Bullied Adolescents. Transl Psychiatry (2018) 8:121. 10.1038/s41398-018-0169-8 29921868 PMC6008402

[113] Dadds MR Moul C Hawes DJ Mendoza Diaz A Brennan J. Individual Differences in Childhood Behavior Disorders Associated With Epigenetic Modulation of the Cortisol Receptor Gene. Child Dev (2015) 86:1311–20. 10.1111/cdev.12391 26152664

[114] de Assis Pinheiro J Freitas FV Borçoi AR Mendes SO Conti CL Arpini JK. Alcohol Consumption, Depression, Overweight and Cortisol Levels as Determining Factors for NR3C1 Gene Methylation. Sci Rep (2021) 11:6768. 10.1038/s41598-021-86189-z 33762648 PMC7990967

[115] Provençal N Arloth J Cattaneo A Anacker C Cattane N Wiechmann T. Glucocorticoid Exposure During Hippocampal Neurogenesis Primes Future Stress Response by Inducing Changes in DNA Methylation. Proc Natl Acad Sci USA (2020) 117:23280–5. 10.1073/pnas.1820842116 PMC7519233 31399550

[116] Constantinof A Boureau L Moisiadis VG Kostaki A Szyf M Matthews SG. Prenatal Glucocorticoid Exposure Results in Changes in Gene Transcription and DNA Methylation in the Female Juvenile Guinea Pig Hippocampus Across Three Generations. Sci Rep (2019) 9:18211. 10.1038/s41598-019-54456-9 31796763 PMC6890750

[117] Kertes DA Kamin HS Hughes DA Rodney NC Bhatt S Mulligan CJ. Prenatal Maternal Stress Predicts Methylation of Genes Regulating the Hypothalamic-Pituitary-Adrenocortical System in Mothers and Newborns in the Democratic Republic of Congo. Child Dev (2016) 87:61–72. 10.1111/cdev.12487 26822443 PMC4733886

[118] Tian FY Rifas-Shiman SL Cardenas A Baccarelli AA DeMeo DL Litonjua AA. Maternal Corticotropin-Releasing Hormone Is Associated With LEP DNA Methylation at Birth and in Childhood: An Epigenome-Wide Study in Project Viva. Int J Obes (2019) 43:1244–55. 10.1038/s41366-018-0249-0 PMC6529291 30464231

[119] Sterrenburg L Gaszner B Boerrigter J Santbergen L Bramini M Elliott E. Chronic Stress Induces Sex-Specific Alterations in Methylation and Expression of Corticotropin-Releasing Factor Gene in the Rat. PloS One (2011) 6:e28128. 10.1371/journal.pone.0028128 22132228 PMC3223222

[120] Levine A Worrell TR Zimnisky R Schmauss C. Early Life Stress Triggers Sustained Changes in Histone Deacetylase Expression and Histone H4 Modifications That Alter Responsiveness to Adolescent Antidepressant Treatment. Neurobiol Dis (2012) 45:488–98. 10.1016/j.nbd.2011.09.005 PMC3225638 21964251

[121] Karen C Rajan KE. Social Behaviour and Epigenetic Status in Adolescent and Adult Rats: The Contribution of Early-Life Stressful Social Experience. Cell Mol Neurobiol (2019) 39:371–85. 10.1007/s10571-019-00655-x PMC11479603 30710320

[122] Dong E Pandey SC. Prenatal Stress Induced Chromatin Remodeling and Risk of Psychopathology in Adulthood. Int Rev Neurobiol (2021) 156:185–215. 10.1016/bs.irn.2020.08.004 33461663 PMC7864549

[123] Zheng Y Fan W Zhang X Dong E. Gestational Stress Induces Depressive-Like and Anxiety-Like Phenotypes Through Epigenetic Regulation of BDNF Expression in Offspring Hippocampus. Epigenetics (2016) 11:150–62. 10.1080/15592294.2016.1146850 PMC4846107 26890656

[124] Lopizzo N Mazzelli M Zonca V Begni V Pariante CM Riva MA. Alterations in Inflammatory Pathways in the Rat Prefrontal Cortex as Early Biological Predictors of the Long-Term Negative Consequences of Exposure to Stress Early in Life. Psychoneuroendocrinology (2021) 124:104794. 10.1016/j.psyneuen.2020.104794 33429258

[125] Vangeel EB Izzi B Hompes T Vansteelandt K Lambrechts D Freson K. DNA Methylation in Imprinted Genes IGF2 and GNASXL Is Associated With Prenatal Maternal Stress. Genes Brain Behav (2015) 14:573–82. 10.1111/gbb.12249 26333472

[126] Cao-Lei L de Rooij SR King S Matthews SG Metz GAS Roseboom TJ. Prenatal Stress and Epigenetics. Neurosci Biobehav Rev (2020) 117:198–210. 10.1016/j.neubiorev.2017.05.016 28528960

[127] Ruiz RJ Gennaro S O’Connor C Dwivedi A Gibeau A Keshinover T. CRH as a Predictor of Preterm Birth in Minority Women. Biol Res Nurs (2016) 18:316–21. 10.1177/1099800415611248 PMC5384468 26512053

[128] Makrigiannakis A Semmler M Briese V Eckerle H Minas V Mylonas I. Maternal Serum Corticotropin-Releasing Hormone and ACTH Levels as Predictive Markers of Premature Labor. Int J Gynecol Obstet (2007) 97:115–9. 10.1016/j.ijgo.2007.01.007 17368647

[129] Wadhwa PD Garite TJ Porto M Glynn L Chicz-Demet A Dunkel-Schetter C. Placental Corticotropin-Releasing Hormone (CRH), Spontaneous Preterm Birth, and Fetal Growth Restriction: A Prospective Investigation. Am J Obstet Gynecol (2004) 191:1063–9. 10.1016/j.ajog.2004.06.070 15507922

